# Predictors of blood pressure and hypertension long-term after treatment of isolated coarctation of the aorta in children—a population-based study

**DOI:** 10.1093/icvts/ivac212

**Published:** 2022-08-09

**Authors:** Mari K Ylinen, Jaana I Pihkala, Jukka T Salminen, Taisto Sarkola

**Affiliations:** Department of Pediatric Cardiology, New Children’s hospital, University of Helsinki and Helsinki University Hospital, Helsinki, Finland; Department of Pediatric Cardiology, New Children’s hospital, University of Helsinki and Helsinki University Hospital, Helsinki, Finland; Department of Pediatric Surgery, New Children’s hospital, University of Helsinki and Helsinki University Hospital, Helsinki, Finland; Department of Pediatric Cardiology, New Children’s hospital, University of Helsinki and Helsinki University Hospital, Helsinki, Finland; Minerva Foundation Institute for Medical Research, Helsinki, Finland

**Keywords:** Coarctation of the aorta, Hypertension, Blood pressure, Surgery, Catheter intervention, Left ventricular mass

## Abstract

**OBJECTIVES:**

The aim of this study was to assess predictors of BP and hypertension and relations between BP and LV mass in a population-based retrospective study of repaired isolated coarctation of aorta.

**METHODS:**

We collected follow-up data until 2018 of 284/304 (93%) patients with coarctation treated by surgery (*n* = 235) or balloon angioplasty/stent (*n* = 37/12) in our unit 2000–2012. Systolic hypertension was defined as systolic BP (SBP) *z*-score ≥+2 standard deviation (SD) or regular use of BP medication. LV hypertrophy was defined as LV mass *z*-score ≥+2 SD or LV mass index g/m^2.7^ ≥95th percentile.

**RESULTS:**

The median (25–75th percentiles) follow-up time and age at follow-up were 9.7 years (6.9–13.2) and 11.8 years (7.9–16.0), respectively. Age at first procedure (*P* = 0.011) and systolic arm-leg-gradient (*P* = 0.007) were positively and transverse arch (*P* = 0.007) and isthmus diameter (*P* = 0.001) *z*-scores at follow-up were negatively associated with SBP *z*-score adjusted for age at follow-up and need for reintervention for coarctation. Systolic hypertension was present in 53/284 (18.7%) and related with increasing age at first procedure (median 33.2 vs 0.6 months; *P* < 0.001) and arm-leg-gradient at follow-up (mean ± SD, −0.3 ± 14.6 vs −6.4 ± 11.6 mmHg; *P* = 0.047) adjusted for reintervention for coarctation and age at follow-up. LV hypertrophy was present in 20/227 (9.3%) and related with SBP *z*-score.

**CONCLUSIONS:**

Higher SBP and hypertension in repaired coarctation of aorta are related with increasing age at first procedure and arm-leg-gradient at follow-up. Transverse arch and isthmus diameters at follow-up are inversely related with SBP.

**Figure:**
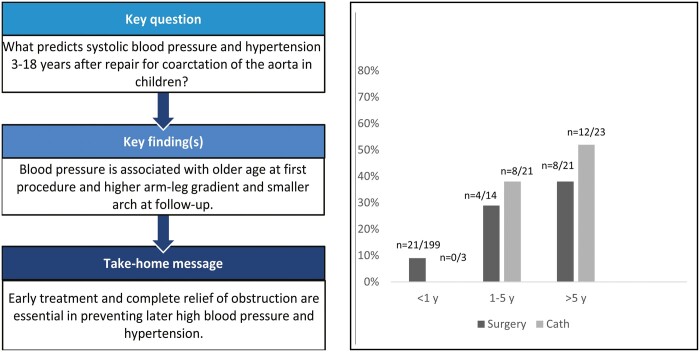

## INTRODUCTION

Repaired isolated coarctation of the aorta (CoA) is commonly related with premature development of high blood pressure (BP) and hypertension in adolescence or during young adulthood [1, 2]. Primary interventions have shifted to infancy and early childhood. Still, a significant proportion of patients are diagnosed later in life. Although surgical relief of the narrowing of the aortic arch is the main modality applied during infancy, percutaneous interventions including balloon angioplasty (BA) with or without stent implantations have become a treatment option in discrete local forms from early childhood. Decision-making regardless of age is influenced by arch and CoA anatomy impacting on the extent of the surgical intervention needed to relieve the obstruction and with potential impact on needs for re-interventions as well as long-term outcomes [3]. Development of recoarctation (re-CoA) is a well-known phenomenon impacting on BP in the upper body of the growing child long term [4–6].

We have previously reported on patient and treatment-related predictors of re-CoA in a population-based study including treatment of all isolated CoAs in Finland between 2000 and 2012 [7]. Furthermore, we have reported BP and intervention-related changes in peripheral artery wall layer thickness and left ventricular (LV) mass among repaired Canadian CoA teenagers [8]. Others have reported, similarly to us, on the role of arch and CoA anatomy and periprocedural treatment and reintervention related predictors of BP in repaired isolated CoA [9]. Longitudinal long-term large population-based contemporary studies comparing BP following surgical or percutaneous treatments of simple native CoAs in relation to arch dimensions are, however, relatively scarce [10].

The aims of the present study were to assess BP, prevalence and treatment of hypertension, explore predictors of BP and hypertension, as well as relations between BP and LV mass long-term in a longitudinal population-based retrospective follow-up study of repaired isolated CoA.

## PATIENTS AND METHODS

### Ethical statement

The ethical review board of the New Children’s Hospital approved the study protocol (308/13/03/03/2014). As the study is register-based, consent of patients was not obtained.

### Study population and design

All children treated for isolated CoA (*n* = 304) at the Children’s Hospital, Helsinki University Hospital, Finland, between 2000 and 2012 were included in this retrospective cohort study. All paediatric cardiac surgery and catheter interventions in Finland are performed at the Children's Hospital in Helsinki making this a population-based study. The institutional criteria for the treatment of isolated native CoA were: (i) critical duct-dependent CoA or 20 mmHg arm-leg systolic BP (SBP) gradient (arm-leg gradient) or (ii) smaller than 0.5 ratio of CoA diameter to aorta at the diaphragm level or (iii) aortic narrowing combined with right arm hypertension. Patients with short segment CoA aged at least 6 months were considered for BA with stent placement considered in older children (>20 kg). The indications for re-CoA intervention were the same as for native CoA intervention. The primary procedures were surgery (*n* = 251), BA (*n* = 40) and stent implantation (*n* = 13). Surgical techniques included end-to-end (*n* = 232), end-to-side (*n* = 3), subclavian flap (*n* = 1) and arch augmentation (*n* = 15) procedures. The median sternotomy was performed in 8 patients. This is an extension of our study concentrating on reintervention rates of isolated CoA [7]. The same cohort was followed up in local central and university hospitals by a paediatric cardiologist or a paediatrician familiar with congenital heart diseases. After primary intervention, the patients were assessed by a cardiologist at 1, 3, 6 and 12 months and thereafter yearly or as considered appropriate. We reviewed the hospital charts for the last available follow-up visits by end of 2018 for these 304 patients including the information of current medication, weight, height, arm and leg BP measurements and echocardiography.

BP measurement at least from the right arm was available in 280/304 (92%), from right arm and from either leg in 258/304 (82%), from both arms in 122/304 (40%) and from all 4 limbs in 115/304 (38%). The recommendation is to assess arm and leg BP at rest in the supine position with oscillometry using appropriately sized cuffs. The mean of 2–3 measurements was used in the analysis after removing outliers. BP gradients were calculated as the difference between right arm and the higher mean of either leg (ankle) values. BP *z*-scores for <18-year-old patients were generated using standard US fourth report data [11] and for 18–29-year-old patients using FinnHealth 2017 data (Finnish Institute for health and welfare: FinnHealth 2017). Body surface area was calculated with the Mosteller formula and used for cardiac and aortic *z*-score calculations. Hypertension was defined as SBP >+2 standard deviation (SD) or use of antihypertensive medication at the latest follow-up visit. Cardiovascular measurements were collected from hospital records as documented by the cardiologist in the clinical setting from high-quality B-mode images obtained in accordance with international guidelines from standard apical, parasternal and suprasternal short and long-axis views (Supplementary Material, Methods).

### Data analyses

Descriptive statistics of data are expressed as medians, means, 25 − 75th percentiles, ranges and SDs [12]. Distribution of continuous variables was evaluated by histograms and statistical significance of normality by Shapiro–Wilks’ test. Surgery, BA or stent groups were analysed separately. Subgroup analyses for children aged ≥6 months at first procedure were performed to compare treatments. Continuous variables associations were assessed using Pearson correlation. Independent sample *t*-test or Mann–Whitney *U* test were used to compare groups. Chi-squared test or Fisher’s exact test was used in cross-tabulation for dichotomous data. Linear regression analysis (enter-method) was used to identify predictors of SBP *z*-score. Variables with significance level *P* < 0.1 in univariate analysis (age at first procedure, age at follow-up, treatment of re-CoA and SBP gradient at follow-up) were included in the model. Body mass index (BMI) *z*-score (multicollinearity with age at first procedure and age at follow-up) was not included, and effects of transverse arch and isthmus *z*-scores were examined separately adjusted for age at first procedure, age at follow-up and treatment of re-CoA. Stepwise-forward (likelihood ratio) logistic regression analysis was performed to analyse associations between different predictors and hypertension. Variables *P* < 0.1 in univariate analyses (age at first procedure, age at follow-up, treatment for re-CoA and systolic arm-leg BP gradient at follow-up) were included in the model as covariates. Mean SBP *z*-scores for treatment groups adjusted for age at first procedure and age at follow-up and mean transverse arch diameter *z*-score change from preintervention to follow-up adjusted for transverse arch diameter *z*-score at preintervention were generated with ANCOVA. All analyses were performed with IBM SPSS Statistics 22.

## RESULTS

In all, follow-up data were available in 284/304 (93%) of the national cohort with 235/251 (94%) available for surgery, 37/40 (93%) for BA and 12/13 (92%) for stent groups as first procedure, respectively. The unadjusted baseline and follow-up characteristics in the 3 treatment groups are shown in Table 1. Patients in the BA and stent groups were significantly older at first procedure, and bicuspid aortic valves were more common among patients with surgery as first procedure. During 2015–2018, 5 additional re-interventions were performed within the study cohort: 3 arch reconstructions in the surgery group (second re-CoA treatment in all 3) and 1 stent procedure for both BA and the stent groups, thus, updated reintervention rates for the original 304 study cohort being 40/251 (16%), 10/40 (25%) and 5/13 (38%), respectively.

**Table 1:** Baseline and follow-up characteristics of the treatment groups stratified by first procedure type

	Surgery, *N* = 235	Balloon angioplasty, *N* = 37	Stent, *N* = 12	*P*-Value/OR (95% CI)
Baseline				
Age at first procedure (years)	0.0 (0.0 to 0.3)	3.0 (1.4 to 7.7)	15.0 (8.8 to 16.3)	<0.001^a^
Sex (male)	161 (68.5%)	25 (67.6%)	9 (75.0%)	0.90^b^
Turner (% of females)	5/74 (5.6%)	0	0	–
Bicuspid aortic valve	99/219 (45.2%)	7/35 (20.0%)	3/10 (30.0%)	0.011^b^
Follow-up				
Age (years)	10.6 (7.2 to 15.1)	16.5 (13.3 to 20.0)	22.1 (15.9 to 24.2)	<0.001^a^
Follow-up time (years)	9.4 (6.8 to 12.8)	12.5 (8.7 to 14.8)	6.5 (5.1 to 10.9)	0.11^a^
Reintervention for re-CoA (cath or surgery, *n* = 304)	40^d^ (15.9%)	10^e^ (25.0%)	5^f^ (38.4%)	0.034^b^
				2.1 (1.0–4.1)
Height (cm)	139.8 (122.6 to 161.9)	170.4 (147.9 to 176.0)	168.5 (151.6 to 182.4)	<0.001^a^
Weight (kg)	33.4 (23.9 to 54.5)	66.7 (39.3 to 78.1)	73.8 (44.1 to 97.0)	<0.001^a^
Body surface area (m^2^)	1.1 (0.6 to 2.5)	1.8 (1.3 to 1.9)	1.9 (1.4 to 2.2)	<0.001^a^
Body mass index *z*-score	0.0 (−0.8 to 0.7)	0.2 (−0.5 to 1.3)	0.4 (0.2 to 2.0)	0.026^a^
Systolic BP (mmHg)	113 ± 13	126 ± 15	132 ± 16	<0.001^c^
Systolic BP (*z*-score)	0.8 ± 0.9	1.2 ± 1.0	1.4 ± 1.6	0.004^c^
Diastolic BP (mmHg)	62 ± 8	65 ± 10	71 ± 13	0.015^c^
Diastolic BP (*z*-score)	0.1 ± 0.8	−0.1 ± 1.0	0.2 ± 0.0	0.483^c^
Systolic arm-leg BP gradient (mmHg)	−6 ± 12	−3 ± 14	9 ± 12	0.044^c^
BP medication	15^g^ (6.4%)	7^h^ (18.9%)	4^i^ (33.3%)	0.002^b^
Hypertension (systolic BP *z*-score >2 or medication)	33 (14.1%)	13 (37.1%)	7 (58.3%)	<0.001^b^
				4.5 (2.3–9.0)
Left ventricular mass (*z*-score)	0.4 ± 1.2	0.8 ± 0.9	−0.4 ± 0.9	0.49^c^
Left ventricular mass (g/m^2.7^)	33.1 ± 8.7	33.3 ± 9.6	27.6 ± 6.2	0.70^c^
Transverse arch diameter *z*-score (*n* = 125/13/4)	−0.5 ± 1.9	−1.3 ± 1.4	1.4 ± 1.5	0.66^c^
Transverse arch diameter *z*-score at preintervention (*n* = 225/34/11)	−1.6 (±2.1)	−0.4 (1.1)	0.2 (±1.5)	<0.001^c^
Transverse arch diameter *z*-score change from preintervention to follow-up (*n* = 118/12/3)	1.1 ± 2.7	−0.6 ± 1.0	0.8 ± 1.9	0.002^c^
Isthmus diameter *z*-score (*n* = 155/17/1)	−1.3 ± 2.1	−1.9 ± 2.0	–	0.94^c^
Isthmus diameter *z*-score at preintervention (*n* = 226/37/12)	−8.9 ± 2.9	−8.4 ± 2.8	−7.2 ± 2.8	0.055^c^
Isthmus diameter *z*-score change from preintervention to follow-up (*n* = 151/17/1)	7.6 ± 3.6	6.6 ± 2.4	–	0.14^c^

Data are provided as median (25–75th percentiles), mean (SD) or *N* (%) and presented as *P*-values or OR and 95% CIs.

a Independent samples Mann–Whitney *U* test, between surgery and cath (balloon angioplasty and stent).

b Pearson Chi-square test, between surgery and cath (balloon angioplasty and stent combined).

c Independent samples *t*-test, between surgery and cath (balloon angioplasty and stent combined).

d Thirty-five balloon angioplasty, 5 surgery.

e Six surgery, 1 balloon angioplasty and 3 stent.

f One surgery, 1 balloon angioplasty and 3 stent.

^g^ Four beta-blocker, 3 Angiotensin-converting enzyme (ACE) inhibitor, 1 Ca-channel blocker, 4 Angiotensin receptor blockers (ARBs), 2 beta-blocker plus ACE inhibitor and 1 Ca-channel blocker plus ARB.

h Three beta-blocker, 3 ACE inhibitor and 1 beta-blocker plus diuretic.

^i^ One ARB, 2 beta-blocker plus ACE inhibitor and 1 beta-blocker plus diuretic.

BP: blood pressure; Cis: confidence intervals; OR: odds ratios; re-CoA: recoarctation; SD: standard deviation.

At follow-up, systolic (114 ± 13.1 vs 115 ± 15.4 mmHg) and diastolic (61 ± 8.6 vs 64 ± 9.6 mmHg) BPs did not differ between males and females. BMI *z*-score was positively associated with SBP *z*-score (*r* = 0.419, *P* = 0.008) and higher in the cath group than in the surgery group (Table 1). BMI *z*-score was also higher in hypertensive compared to non-hypertensive (Table 2). SBP and diastolic BP values were higher in the BA and stent groups compared with surgery group (Table 1).

**Table 2:** Baseline and follow-up characteristics of the treatment groups stratified by presence of hypertension (systolic blood pressure *z*-score >2 or use of blood pressure medication)

	Hypertension, *N* = 53	No hypertension, *N* = 231	*P*-Value/OR (95% CI)
Age at first procedure (years)	2.8 (0.2 to 9.3)	0.1 (0.0 to 0.5)	<0.001^a^
Surgery as first procedure	33	201	<0.001^c^
Cath (BA or stent) as first procedure	20	27	4.5 (2.3–9.0)
Reintervention for re-CoA (cath or surgery)	14/53 (26.4%)	36/231 (15.6%)	0.068^c^
			1.9 (0.9–3.9)
Age at follow-up (years)	16.3 (10.6 to 20.3)	10.8 (7.3 to 15.3)	<0.001^a^
Follow-up time (years)	9.6 (7.8 to 13.9)	9.7 (6.7 to 12.9)	0.26^a^
BMI *z*-score	0.6 (−0.2 to 1.3)	−0.1 (−0.8 to 0.6)	0.001^a^
Systolic BP (mmHg)	134.5 ± 13.2	110.8 ± 11.0	<0.001^b^
Systolic BP (*z*-score)	2.2 (±0.9)	0.6 (±0.8)	<0.001^b^
Diastolic BP (mmHg)	68.2 ± 11.8	60.8 ± 7.5	<0.001^b^
Diastolic BP (*z*-score)	0.3 (±1.1)	0.0 (±0.7)	0.055^b^
Systolic arm-leg BP gradient at follow-up (mmHg)	−1.9 ± 13.9	−6.5 ± 11.6	0.024^b^
Left ventricular mass (*z*-score)	0.6 ± 1.1	0.4 ± 1.2	0.36^b^
Left ventricular mass (g/m^2.7^)	34.9 ± 7.2	32.8 ± 8.7	0.18^b^
Transverse arch diameter *z*-score at follow-up, *N* = 20/120	−1.0 ± 2.2	−0.5 ± 1.8	0.23^b^
Isthmus diameter *z*-score at follow-up, *N* = 21/151	−1.8 ± 2.4	−1.4 ± 2.1	0.35^b^
Transverse arch diameter *z*-score at preintervention, *N* = 49/218	−1.0 ± 2.0	−1.5 ± 2.0	0.12^b^
Isthmus diameter *z*-score at preintervention, *N* = 53/219	−8.7 ± 3.6	−8.8 ± 2.7	0.85^b^
Change in transverse aortic arch *z*-score from preintervention to follow-up, *N* = 17/115	0.4 ± 3.1	1.1 ± 2.5	0.30^b^
Change in isthmus *z*-score from preintervention to follow-up, *N* = 21/147	6.5 ± 4.6	7.6 ± 3.3	0.27^b^

Data is provided as median (25–75th percentiles), mean (SD) or *N* (%) and presented as *P*-values or OR (odds ratios) and 95% CIs (confidence intervals).

a Independent samples Mann–Whitney *U* test.

b Independent samples *t*-test.

c Pearson Chi-square test.

BA: balloon angioplasty; BMI: body mass index; BP: blood pressure; CI: confidence interval; OR: odds ratio; re-CoA: recoarctation.

In all, 26/284 (9.2%) of patients were on BP medication at follow-up (Table 1). BP medication was more prevalent following BA 7/37 (18.9%) and stent 4/12 (33.3%) compared with surgery 15/235 (6.4%) as primary procedure (*P* = 0.002). Prevalence of systolic hypertension (SBP *z*-score >+2 SD) was 34/280 (12.1%) for all groups combined, and 23/234 (9.8%) for surgery, 6/34 (17.6%) for BA and 5/12 (41.7%) for stent groups (*P* = 0.003). The prevalence of combined systolic hypertension or regular antihypertensive medication at follow-up was 53/284 (18.7%) for all groups combined, and 33/234 (14.1%) for surgery, 13/35 (37.1%) for the BA group and 7/12 (58.3%) for the stent group (*P* < 0.001). Diastolic hypertension (>+2 SD) was rare and found only in 6 patients (3 with systolic hypertension).

**Figure 1:**
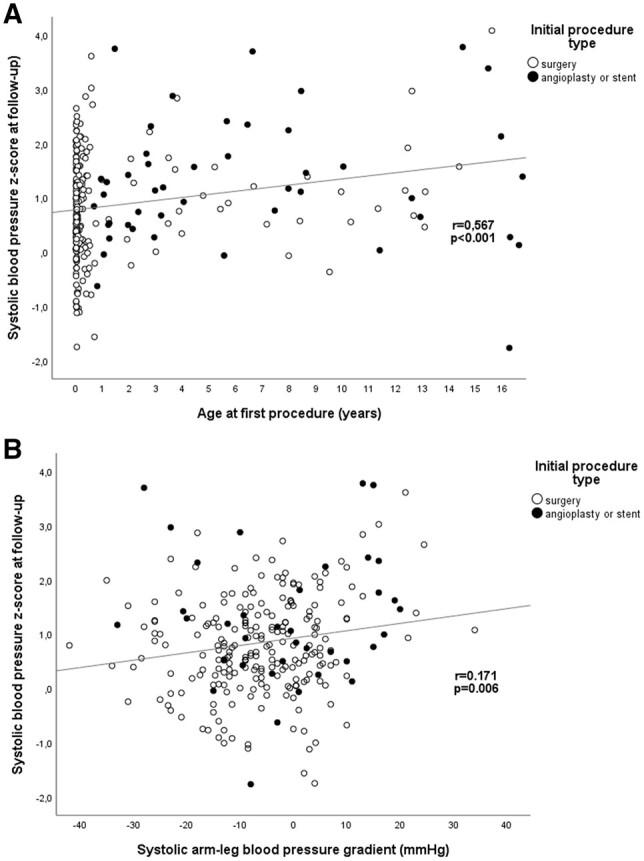
Associations between age at first procedure and systolic blood pressure (**A**) and systolic arm-leg-blood pressure-gradient and systolic blood pressure (**B**) at follow-up.

SBP *z*-score was positively associated with age at follow-up (*r* = 0.231, *P* < 0.001), and hypertension at follow-up was more common in older age groups for both surgery and cath groups (in 11/115, 9.6% in <10 years and in 42/166, 25.3% in ≥10 years). There was a significant positive association between age at primary CoA repair and SBP at follow-up (*r* = 0.567, *P*  < 0.001; Fig. 1A). Patients with hypertension at follow-up were significantly older at primary procedure compared with patients without hypertension (Table 2). Furthermore, 18/188 (9.6%) of patients with primary procedure at <6 months of age had hypertension at follow-up compared to 35/93 (37.6%) of those who had first procedure at the age of ≥6 months [odds ratio (OR) 5.7, 95% confidence interval (CI) 3.0–10.8]. SBP *z*-scores did not differ between arch augmentation (*n* = 14) and end-to-end repair as first procedure (*n* = 220) (0.8 ± 1.0 vs 0.8 ± 0.9; *P* = 0.80). Hypertension was more common following arch augmentation (*n* = 5/14, 35.7% vs 28/220, 12.7%; OR 3.8, 95% CI 1.2–12.2), but this was explained by older age at first procedure (median 45.5 vs 0.5 months; *P* = 0.043).

Systolic arm-leg BP-gradient was significantly correlated with SBP *z*-score in all patients (*r* = 0.171, *P* = 0.006; Fig. 1B). A significant difference in arm-leg-BP-gradient was observed for surgery, BA and stent groups. This difference was reflected in the significant difference in SBP *z*-score (and hypertension) between treatment groups, with stented patients showing highest SBP and surgery patients lowest SBP at follow-up (Tables 1 and 2). This difference in SBP *z*-score disappeared when adjusting for age at first intervention and age at follow-up, adjusted means and 95% CIs, 0.9 (0.7–1.0), 1.2 (0.9–1.5) and 0.7 (0.0–1.4) for surgery, BA and stent groups, respectively (*P* = 0.15).

The SBP gradient between right and left arm did not differ significantly between the 3 treatment groups (data not shown). SBP *z*-scores (1.1 ± 1.1 vs 0.8 ± 1.0; *P* = 0.062), systolic arm-leg BP gradient (−5.7 ± 12.5 vs −5.8 ± 12.0 mmHg; *P* = 0.97) and the presence of hypertension (12/50 vs 39/231, OR, 95% CI 1.9, 0.9–3.9) at follow-up was similar among patients with and without reintervention for re-CoA.

**Figure 2:**
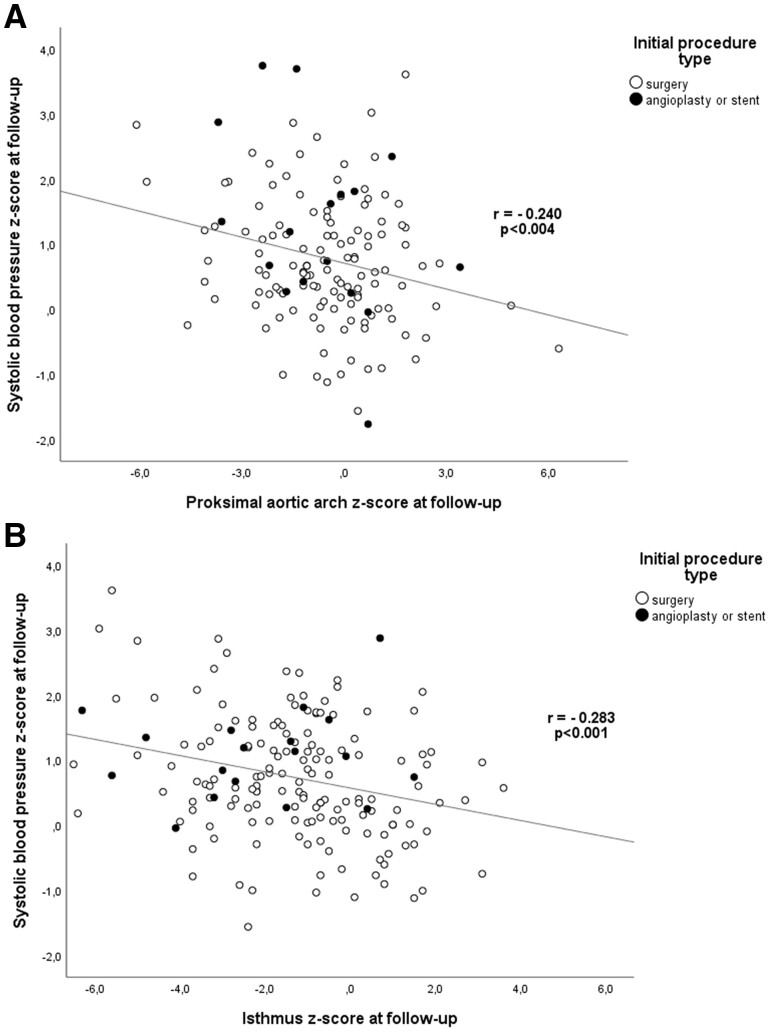
Associations between transverse arch diameter (**A**) and isthmus diameter (**B**) and systolic BP at follow-up.

There was a significant negative correlation between both transverse aortic arch as well as isthmus *z*-score and right arm SBP *z*-scores at follow-up (*r* = −0.240, *P* = 0.004 and *r* = −0.283, *P*  < 0.001, respectively, Fig. 2A and B). However, there was no statistically significant difference between arch and isthmus diameter *z*-scores at follow-up, or in corresponding preintervention values at first procedure, among patients with and without hypertension at follow-up (Table 2).

Transverse arch diameter *z*-score change from preintervention to follow-up (i.e. growth in relation to body size) was significantly smaller following BA compared with surgery as first procedure (Table 1), but this was not reflected in presence of hypertension (Table 2). This difference in transverse arch diameter *z*-score change disappeared when adjusting for the preintervention transverse arch diameter [adjusted mean and 95% CI, 1.0 (0.7–1.4), 0.0 (−1.0 to 1.1) and 2.1 (−0.1 to 4.2) for surgery, BA and stent groups] (*P* = 0.12).

**Figure 3:**
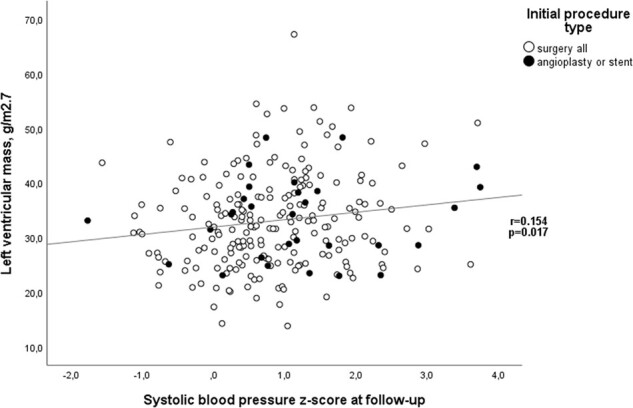
Association between systolic blood pressure and left ventricular mass at follow-up.

LV hypertrophy was present in 20/227 (8.8%) using the ≥2 *z*-score criteria and in 12/239 (5.0%) using the >95th percentile criteria. There was no significant correlation between systolic (or diastolic) BP *z*-score and LV mass *z*-score (data not shown). However, there was a positive correlation between SBP *z*-score and LV mass g/m^2.7^ (*r* = 0.154, *P* = 0.027; Fig. 3). LV mass *z*-score and LV mass g/m^2.7^ were not statistically different between surgery and cath groups (Table 1) nor between hypertension and no hypertension groups (Table 2). LV hypertrophy, defined by LV mass *z*-score ≥2 SD or LV mass g/m^2.7^ ≥95 percentile, was significantly associated with higher SBP *z*-score (1.3 ± 1.2 vs 0.8 ± 1.0, *P* = 0.040, and 1.4 ± 0.8 vs 0.8 ± 1.0, *P* = 0.040, respectively).

In subanalyses, comparing surgical (*n* = 46) and percutaneous treatments (*n* = 49) in children treated at ≥6 months of age, the groups differed significantly only regarding higher reintervention rates for re-CoA in the cath group (Supplementary Material, Table S1). There was a tendency for higher systolic arm-leg gradient in the cath group.

In a stepwise linear regression analysis, independent predictors of higher SBP *z*-score were increasing age in years at first procedure (*B* = 0.060, *P* = 0.011) and higher systolic arm-leg BP gradient in mmHg (*B* = 0.013, *P* = 0.007, *R*^2^ = 0.045). Transverse aortic arch diameter (*B* = −0.120, *P* = 0.012, *R*^2^ = 0.090) and isthmus diameter (*B* = −0.089, *P* = 0.011, *R*^2^ = 0.099) *z*-scores at follow-up were negatively associated with SBP *z*-score. Independent predictors of hypertension, explored by stepwise logistic regression analysis, included younger age in years at first procedure (*B* = 0.152, OR 1.2 95% CI 1.1–1.3, *P* = 0.001) and higher systolic arm-leg BP gradient in mmHg (*B* = 0.029, OR 1.0 95% CI 1.0–1.06, *P* = 0.047) at follow-up (*R*^2^ = 0.103).

## DISCUSSION

In the present national population-based single-centre study systolic hypertension was present in 18.7% at median 10 years after treatment for native isolated CoA. Hypertension increased with age and was found in 9.6% <10 years, 15.5% 10–16 years and 39.1% >16 years age groups. Older age at primary CoA repair, higher SBP gradient and smaller arch diameter at follow-up predicted higher SBP and hypertension. LV hypertrophy was related with higher SBP. The study shows that early treatment and complete relief of obstruction prevent later high BP.

CoA, often regarded as a simple heart defect, is still related with reduced life expectancy and increased mortality due to cardiovascular complications linked with elevated BP and other cardiovascular risk factors [13–15]. Hypertension in patients with repaired CoA is multifactorial and related with anatomical residual arch gradients as well as alterations in renin–angiotensin and baroreceptor systems with combined long-term impacts on arterial wall and LV mass [8].

Our median 10-year hypertension prevalence is lower than the 35% reported in a systematic review [16]. We defined systolic hypertension based on office BP measurements or use of BP medication. Studies including ambulatory BP assessments report systematically more hypertension in patients with repaired CoA [10, 16]. The criteria to start BP medication in children in Finland are relatively conservative (stage 1–2 hypertension based on follow-up), which may have influenced our results. In the present study, the relationship between age at first procedure and SBP *z*-score at follow-up was linear. The risk for later hypertension was found in children operated at more than 6 months of age compared to those operated earlier (38% vs 10%). Our relatively young age at follow-up and high proportion of patients operated during the newborn period may then explain prevalence differences with previous studies.

The international criteria for the arm-leg-BP gradient to treat CoA and re-CoA are 20 mmHg [17]. In the present cohort, a gradient of ≥20 mmHg at follow-up was found in 6 patients only. In follow-up studies, residual obstruction has been associated with systolic hypertension [10, 18]. Our study was consistent with this showing systolic arm-leg BP gradient to be directly linked with SBP. Thus, patients with mild residual gradients, but not considered sufficient for reintervention, may still have elevated SBP [1, 19]. Previous studies show that mild arch hypoplasia, based on magnetic resonance measurements, without significant arm-leg-gradient is associated with systemic hypertension 14 years after surgical or percutaneous CoA treatment [5]. In the present similar but younger large study cohort, transverse aortic arch diameter was similarly negatively associated with SBP and without an association between arch diameter and arm-leg-gradient. Similarly, a study from Canada reported hypertension after stented native and recurrent CoA related with relative arch hypoplasia but without significant arch gradient [20]. The present study adds to this by showing an association between isthmus diameter, arm-leg BP gradient and SBP suggesting an anatomical causal contribution for elevated SBP in the preductal region. It has been suggested that exposition of the proximal arterial tree to elevated BP may change its structure and decrease baroreceptor sensitivity that in combination could increase arterial stiffness systolic and BP further [21–24]. Among patients with native CoA treated primarily with BA and/or stent, older age at first procedure and significantly higher prevalence of re-interventions are both related with longer high SBP exposure, which likely explains the higher SBP and higher hypertension prevalence in these groups. Overall, these results add to the increasing evidence suggesting a central role of any arch narrowing in the development of elevated preductal BP and hypertension in repaired isolated CoA.

LV hypertrophy is related with increased afterload. The present study found LV hypertrophy in 9% of patients at the relatively young median age 12 years. We were also able to show LV hypertrophy to be linked with SBP, which was also linked with increasing BMI, and thus cardiovascular risk overall. Previous studies applying similar criteria reported systolic hypertension associated LV hypertrophy in 55% of patients at 24 years following CoA repair [18]. Another study reported LV hypertrophy in 32% of patients, a small difference in LV mass between hypertensive and normotensive patients (38.1 vs 35.0 g/m^2.7^, respectively) at median 8.5 years from CoA repair, and without significant associations with SBP [10]. Taken together, the literature suggests a link between early development of high SBP increasing LV mass over time which add to long-term cardiovascular morbidity in CoA patients.

###  

Main study limitations relate to the retrospective register-based study design. Office BP values at follow-up were only available and we were not able to include 24-h ambulatory and exercise BP data. Previous studies show masked hypertension in 15% of office BP normotensive CoA patients [10]. On the other hand, unlike in many other studies, lower limb BP data were included in the present study. The population-based large sample and low attrition rate at follow-up are also significant strengths.

The present study reports early systolic hypertension in a considerable proportion of young repaired isolated CoA patients compared with contemporary BP data from the healthy Finnish population [25]. Older age at CoA repair, residual arm-leg BP gradient and smaller aortic dimensions predict higher SBP that is reflected in increased LV mass. Early detection and treatment as well as complete relief of arch obstruction are essential in preventing early hypertension and related cardiovascular morbidity long term in children with isolated CoA.

## SUPPLEMENTARY MATERIAL

Supplementary material is available at *ICVTS* online.

## Supplementary Material

ivac212_Supplementary_Data

## Data Availability

The data underlying this article will be shared on reasonable request to the corresponding author.

## Notes

Presented at the 54th annual meeting of the Association for European Paediatric and Congenital Cardiology (AEPC), Gothenburg, Sweden, 25–27 May 2021.

## ABBREVIATIONS

BA: Balloon angioplasty
BMI: Body mass index
BP: Blood pressure
CI: Confidence interval
CoA: Coarctation of the aorta
LV: Left ventricular
OR: Odds ratio
re-CoA: Recoarctation
ROC: Receiver operating characteristic curve
SBP: Systolic blood pressure
SD: Standard deviation
