# Supplementary material for: Predictors of blood pressure and hypertension long-term after treatment of isolated coarctation of the aorta in children—a population-based study
Source: Interact Cardiovasc Thorac Surg. 2022 Aug 9;35(3):ivac212. doi: 10.1093/icvts/ivac212 (PMC9380783; doi:10.1093/icvts/ivac212)
Supplement: ivac212_Supplementary_Data [file ivac212_supplementary_data.docx]

**Supplemental Material to article Ylinen et al Predictors of blood pressure and hypertension long-term after treatment of isolated coarctation of the aorta in children - A population-based study.**

**Supplemental methods description of cardiovascular measurements**

Cardiovascular measurements were collected from hospital records as documented by the cardiologist in the clinical setting from high-quality B-mode images obtained in accordance with international guidelines from standard apical, parasternal and suprasternal short and long-axis views [1,2]. Proximal transverse arch (later transverse arch) and isthmus dimensions were in the reports obtained from the suprasternal long-axis view according to guidelines. Missing information in reports was explored by analysis of digitally stored cine clips when available. Z-scores were generated with a calculator for healthy children developed by The Hospital for Sick Children, Toronto, Canada. The calculator (unpublished) has been well validated clinically over the last 20 years and is comparable to the Boston z-scores for most of the parameters. LV mass was calculated based on M-mode measurements using the Devereux’ formula [3]. LV mass z-scores for <18-year-old were generated based on calculated lean body mass [4] and for 18–29-year-old based on LV mass indexed by body surface area (g/m^2^) using NORRE study normal data [5]. LV hypertrophy was defined as LV mass z-score ≥2 SD. LV mass was in addition indexed for height and analyzed separately as g/m^2,7^ with LV hypertrophy defined as >95^th^ percentile [6].

References

[1] Lai WW, Geva T, Shirali GS, Frommelt PC, Humes RA, Brook MM et al. Guidelines and standards for performance of a pediatric echocardiogram: a report from the Task Force of the Pediatric Council of the American Society of Echocardiography. J Am Soc Echocardiogr 2006;19:1413-30.

[2] Lopez L, Colan SD, Frommelt PC, Ensing GJ, Kendall K, Younoszai AK et al. Recommendations for quantification methods during the performance of a pediatric echocardiogram: a report from the Pediatric Measurements Writing Group of the American Society of Echocardiography Pediatric and Congenital Heart Disease Council. J Am Soc Echocardiogr 2010;23:465-95; quiz 576-7.

[3] Devereux RB, Alonso DR, Lutas EM, Gottlieb GJ, Campo E, Sachs I et al. Echocardiographic assessment of left ventricular hypertrophy: comparison to necropsy findings. Am J Cardiol 1986;57:450-8.

[4] Foster BJ, Platt RW, Zemel BS. Development and validation of a predictive equation for lean body mass in children and adolescents. Ann Hum Biol 2012;39:171-82.

[5] Kou S, Caballero L, Dulgheru R, Voilliot D, De Sousa C, Kacharava G et al. Echocardiographic reference ranges for normal cardiac chamber size: results from the NORRE study. Eur Heart J Cardiovasc Imaging 2014;15:680-90.

[6] de Simone G, Daniels SR, Devereux RB, Meyer RA, Roman MJ, de Divitiis O et al. Left ventricular mass and body size in normotensive children and adults: assessment of allometric relations and impact of overweight. J Am Coll Cardiol 1992;20:1251-60.

**SUPPLEMENTAL TABLE 1**. Characteristics of study subjects (with follow-up data available) in surgery and percutaneous groups in children aged ≥6 months stratified by first procedure type.

| Characteristics | Surgery  (n=46) | Percutaneous (n=49) | P-value/ OR (95%CI) |
| --- | --- | --- | --- |
| Age at first procedure (years, median, range) | 3.9 (1.2-10.0) | 5.6 (2.0-9.4) | 0.40 ^c^ |
| Sex (male, %) | 29 (63.0%) | 34 (69.4%) | 0.51 ^e^ |
| Reintervention for reCoA (≥6 months in the whole cohort) | 1 (2%) ^a^ | 15 (28%) ^b^ | <0.001 ^e^  18.9 (2.4-149.9) |
| Age at follow-up (years, median, range) | 15.5 (11.6-18.0) | 17.4 (14.1-21.2) | 0.088 ^c^ |
| Body mass index (Z-score) | 0.2 (-0.5-0.8) | 0.3 (-0.2-1.3) | 0.34 ^c^ |
| Systolic BP (Z score) | 1.1±1.2 | 1.3±1.2 | 0.52^d^ |
| Diastolic BP (Z-score) | 0.1±1.0 | -0.1±1.0 | 0.55 ^d^ |
| Systolic arm-leg BP gradient at follow-up | -7.1±14.1 | -1.4±14.0 | 0.084 ^d^ |
| Hypertension (n, %) | 15 (32.6%) | 20 (42.6%) | 0.39 ^e^  1.5 (0.7-3.6) |
| Transverse arch diameter Z-score at follow-up, n=20/17 | -1.0±2.0 | -0.7±1.8 | 0.66 ^d^ |
| Isthmus diameter Z-score at follow-up, n= 22/18 | -1.9±2.3 | -2.1±2.2 | 0.74 ^d^ |
| Change in transverse aortic arch Z-score from preintervention to follow-up, n=18/5, | -0.3±2.4 | -0.3±1.3 | 0.98 ^d^ |
| Change in isthmus diameter Z-score from preintervention to follow-up, n=18/18 | 7.1±4.7 | 6.3±2.6 | 0.50 ^d^ |
|  |  |  |  |
| Left ventricular mass (Z-score), n=35/30 | 0.5±1.5 | 0.6±1.0 | 0.70 ^d^ |
| Left ventricular mass (g/m ^2,7^), n=35/30 | 32.6±10.8 | 33.5±7.3 | 0.72 ^d^ |

Data is provided as median (25-75^th^ percentiles), mean (SD) or N (%) and presented as P-values or OR (odds ratios) and 95% CIs (confidence intervals)

^a^ Includes 1 surgery, the whole Surgery cohort ≥6 months, n=49 (41 end-to-end anastomosis, 2 prosthesis, and 6 arch augmentation as first intervention)

^b^ Includes 7 surgery, 2 balloon angioplasty and 6 stent procedures, the whole percutaneus cohort ≥6 months, n=53 (13 stent and 40 balloon angioplasty as first intervention)

^c^ Mann-Whitney U-test

^d^ Independent sample T-test

^e^ Pearson Chi-square test/ Fisher`s exact test
